# FPGA accelerator for protein secondary structure prediction based on the GOR algorithm

**DOI:** 10.1186/1471-2105-12-S1-S5

**Published:** 2011-02-15

**Authors:** Fei Xia, Yong Dou, Guoqing Lei, Yusong Tan

**Affiliations:** 1 National Laboratory for Parallel&Distributed Processing, Department of Computer Science, National University of Defense Technology, ChangSha, 410073, China

## Abstract

**Background:**

Protein is an important molecule that performs a wide range of functions in biological systems. Recently, the protein folding attracts much more attention since the function of protein can be generally derived from its molecular structure. The GOR algorithm is one of the most successful computational methods and has been widely used as an efficient analysis tool to predict secondary structure from protein sequence. However, the execution time is still intolerable with the steep growth in protein database. Recently, FPGA chips have emerged as one promising application accelerator to accelerate bioinformatics algorithms by exploiting fine-grained custom design.

**Results:**

In this paper, we propose a complete fine-grained parallel hardware implementation on FPGA to accelerate the GOR-IV package for 2D protein structure prediction. To improve computing efficiency, we partition the parameter table into small segments and access them in parallel. We aggressively exploit data reuse schemes to minimize the need for loading data from external memory. The whole computation structure is carefully pipelined to overlap the sequence loading, computing and back-writing operations as much as possible. We implemented a complete GOR desktop system based on an FPGA chip XC5VLX330.

**Conclusions:**

The experimental results show a speedup factor of more than 430x over the original GOR-IV version and 110x speedup over the optimized version with multi-thread SIMD implementation running on a PC platform with AMD Phenom 9650 Quad CPU for 2D protein structure prediction. However, the power consumption is only about 30% of that of current general-propose CPUs.

## Introduction

Protein is an important molecule that performs a wide range of functions in biological systems. The function of a protein molecule generally can be derived from its tertiary structure. Currently, the most structures are determined by X-ray diffraction and protein nuclear magnetic resonance (NMR). However, these methods are time consuming and very expensive. With the exponential growth of protein sequence database, such as the EMBL-EBI protein database (UniProKB/TrEMBL), which has doubled in size every 18*~*24 months for the last decade and now it contains 10,867,798 sequence entries, comprising 3502326038 amino acids (protein sequence growth in UniProtKB [1]). However, the number of known structures in the Protein Data-Base (PDB) is just more than 65,000 at present (protein structure growth in PDB [2]). The gap between the number of known protein sequences and structures in PDB continuously grows at an incredible rate. Therefore, the prediction of protein structure and function from amino acid sequence by computational method becomes a most important problem in modern molecular biology and bioinformatics. The prediction of tertiary structure is one of the ultimate goals of protein science. Some methods, such as the homology modeling [3], protein fold recognition [4] or ab initio modeling [5] have been presented for protein 3-Dimensional structure prediction from amino acid sequence directly, but those methods are too complex and unfeasible in some conditions. Instead of predicting the full 3D structure directly, it is much easier to predict fundamental elements of the secondary structure of proteins: a-helices, b-sheets, coils, and turns. All these elements can be easily observed in protein 3D structures and this 2D structure knowledge can serve as an input for protein tertiary structure prediction successfully.

Several approaches have been developed for predicting 2D structure from amino acid sequence: the Chou-Fasman [6], GOR (Garnier-Osguthorpe-Robson) method [7][8], Hydrophobic-Polar (HP) model [9] and some artificial intelligence methods such as neural networks [10], machine learning [11], combine approaches [12] and nearest neighboring methods [13]. The GOR algorithm is one of the earliest and most successful method for secondary structure prediction from protein sequence. It is based on the information theory combined with the Bayesian statistics. Although predicting protein 2D structure using the GOR approach is efficient in the classical sense, the execution time is still intolerable with the steep growth in protein database. For example, scanning of an input protein database with 88,448,646 amino acids on an AMD Phenom 9650 Quad CPU would take about 390s (6.5 minutes) and would take more than 4300s (about 1.2 hours) to scan a PDB with 1,207,406,759 residues using the GOR-IV software package.

## Related works

To our knowledge, there is no parallel implementation of the GOR algorithm running on general-purpose CPUs or GPU platforms for protein 2D structure prediction at present. However, high performance parallel computers based on general-purpose microprocessors are widely used to accelerate bioinformatics applications such as pairwise/multiple sequence alignment, database searching and RNA secondary structure prediction.

Kuo-Bin Li [14] explored a parallel approach to accelerate ClustalW using MPI (Message Passing Interface) on traditional parallel computers in 2002. This implementation achieves a speedup of 4.3x using 16 processors. In [15], Christopher Dwan et al. presented a parallel implementation of the ClustalW package on the SGI Altix XE Cluster with 32 CPUs. They reported the speedups of 7.2x and 17.1x for DNA and protein alignment, respectively. Yu-Lun Kuo et al. [16] implemented parallel mpiBLAST (BLAST, Basic Local Alignment Search Tool) for database searching application on a symmetric multiprocessors (SMP) cluster which consists of 16 CPUs. The paper [15] also introduced a parallel implementation for accelerating the BLASTp algorithm for protein database searching and they reported a 23.6x speedup over the NCBI BLAST version for searching 1,000 protein sequences against the ”NR” dataset from NCBI, which consists of approximately 2GB of amino acid sequences. Large-scale supercomputers like the IBM Blue Gene/L are also used to accelerate the BLAST algorithm [17]. The system consisted of 4096 nodes, where each node consisted of two 700 MHz PowerPC 440D processors and nearly linear speedups can be achieved for large-scale database searching.

For RNA secondary structure prediction, Tan G. et al. presented a parallel implementation of the Zuker algorithm based on the MFE (minimal free energy) model on PC cluster for single RNA sequence. They report a 19x speedup on a 32-processor system, DAWNING 4000 [18], and 8x on a cluster with 16 Opteron processors running at 2.2GHz, each with 3GB RAM [19]. In 2005, T. Liu et al. presented a parallel CYK/inside algorithm between 3D matrix layers on two PC cluster systems [20]. The implementation achieves a speedup of 16x using 20 2.0GHz Xeon CPUs on the PC cluster and a speedup of 36x using 48 1.0GHz Alpha EV68 processors on the cluster of SMPs. Parallel efficiency on CPU platform is greatly limited by the fine-grained bit-wise operations, complicated data dependency and tight synchronization. Thus, efficiently executing the bioinformatics applications on a general-purpose computer or a multi-core architecture becomes very awkward. Moreover, the use, maintenance and management costs of large scale parallel computer systems are very high. High performance parallel computers are too expensive for many research institutes to use easily.

Recently, the use of FPGA (Field Programmable Gate-Array) coprocessors has become a promising approach for accelerating bioinformatics applications. The computational capability of FPGAs is increasing rapidly. The top level FPGA chip from Xilinx Virtex 6 series contains 118560 slices, 38304 Kbits storage and more than 2000 DSP modules. However, its power consumption is less than 30W. Additionally, the reconfigurability of FPGA chips also enables algorithms to be implemented with different computing structures on the same hardware platform. The possibility of using a combination of FPGAs and general-purpose CPUs to accelerate bioinformatics application has attracted much more attention. To the best of our knowledge, there is no parallel FPGA implementation for accelerating the GOR algorithm at present. However, Nilton B. Armstrong et al. [21] implemented parallel 2D Hydrophobic-Polar (HP) model on Altera FPGA EP2S15F484C3 for 2D structure prediction. In 2009, Advait Jain et al. [22] presented a parallel implementation of the Bhageerath [23] package for 3D structure prediction based on FPGA chip and they achieved 5-fold speedup over software version on general-purpose CPU.

In this paper, we propose a complete fine-grained parallel hardware implementation on FPGA to accelerate the GOR-IV package for 2D protein structure prediction. For improving computing efficiency, we partition the parameter table into small sections and access them in parallel. We aggressively exploit data reuse schemes to minimize the need for loading data from external memory. The whole computation structure is carefully pipelined in order to overlap sequence load, computing and back-writing operations as much as possible. We implemented the complete GOR algorithm accelerator on a single FPGA chip (XC5VLX330). The experimental results show a factor of more than 430x speedup over the GOR-IV software version and 110x speedup over the optimized version with multi-thread SIMD implementation running on a PC platform with AMD Phenom 9650 Quad CPU for folding a protein dataset with 6,028,192 sequences, 1,207,406,759 amino acids. However, the power consumption is only about 30% of that of the current general purpose CPUs.

## Overview of the GOR algorithm

The GOR program is one of the first major methods proposed for protein secondary structure prediction from sequence. The original version (GOR-I) was released in 1978 by Garnier, Osguthorpe and Robson. The basic idea of the GOR method is the use of the information theory-based and Bayesian statistics method to relate the amino acid sequence to the protein secondary structure [24]. It takes into account not only the propensities of individual amino acids to form particular secondary structures, but also the conditional probability of the amino acid to form a secondary structure given that its immediate neighbors have already formed that structure [25].

In the past twenty years, the GOR method has been improved by adopting larger structure databases and more exact statistical model for computing information function. The GOR-IV analyzes sequences to predict alpha helix, beta sheet, turn, or random coil secondary structure at each position based on 17-amino acid sequence windows to consider the information of local segment. Eight nearest neighboring residues on each side are considered for a given residue and a database of 267 sequences with known secondary structure to calculate the information function. The latest software version is GOR-V, which is online at the web based protein secondary structure internet prediction server [26]. The most crucial change was the inclusion of evolutionary information using PSI-BLAST [27] to increase the information content for improved discrimination among secondary structures, which combines information theory, Bayesian statistics and evolutionary information. It reaches (with the full jack-knife procedure) an accuracy of prediction Q3 of 73.5%.

After a careful survey, we chose the GOR-IV as the candidate for fine-grained parallel implementation. The kernel recursions, relationship and connotation in this program have been explicated in detail from a viewpoint of information theory and statistics algorithm by [7], [8]. In this study, we will give a characteristic analysis of the GOR-IV in fine-grained parallelism and hardware acceleration aspect.

The GOR runs with a single protein sequence as input, the kernel of the algorithm executes in three steps. First, it predicts the 2D structure of the input protein sequence based on the information theory combined with the Bayesian statistics. For each input amino acid, it computes three probability values of fundamental conformation and selects the largest one for judging the current amino acid belongs to which one of the three secondary structure elements of protein: helix (H), extended(b-sheet) (E), or coil (C). The latter two stages perform a scanning procedure to correct the secondary structure generated by the first stage. The GOR-IV gives the output consists of the protein sequence and the predicted secondary structure in rows, H=helix, E=extended or beta strand and C=coil with the probability values for each secondary structure at each amino acid position. Our study shows most of the execution time, over 99%, is spent in the first stage (the *Predic* function). Therefore, how to execute the *Predic* function quickly is critical to accelerate the GOR program.

## Methods

### System architecture

**Figure 1:**
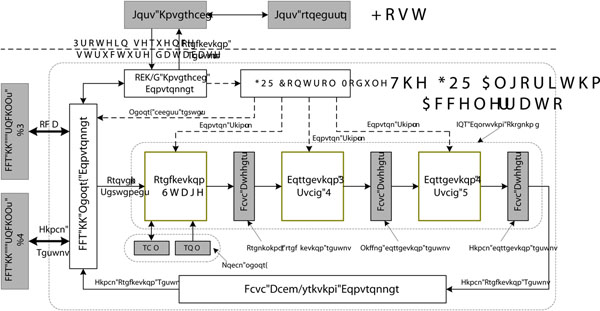
**The structure of the GOR algorithm accelerator.** This figure describes the structure of GOR algorithm accelerator. The accelerator comprises one FPGA chip (Virtex5 XC5VLX330), two DDRII modules, and a PCI-E*×*8 interface to the host PC.

Our protein 2D structure prediction platform consists of a reconfigurable algorithm accelerator and a host PC. The accelerator receives input protein data stream of length *N* with 5-bit binary encoding and a database of 267 sequences with known secondary structure (for computing information function), then executes the 2D structure prediction and correction and returns prediction results to the host for display. The structure is shown in Figure 1.

The accelerating engine comprises one FPGA chip (Virtex5 XC5VLX330), two DDRII modules, and a PCI-E*×*8 interface to the host PC. Two DDRII SODIMMs store Protein Data-Base (PDB) and prediction results respectively, which are connected to the FPGA pad directly, and the memory controller is implemented in FPGA chip. The PCI-E interface is responsible for transferring the initial data (protein sequence database and secondary structure database), the configure commands (start and interrupt signals) and the final results between the accelerator and the host. The effective bandwidth reaches 1GB/s. The core of the GOR algorithm accelerator is composed of a GOR Control Module, a GOR Computing Pipeline and a Data Back-writing Controller. The GOR Control Module is responsible for initializing the Computing Pipeline, assigning protein sequence dynamically to the Computing Pipeline. The Data Back-writing Controller is responsible for buffering protein sequence and the predicted secondary structure and writing them back to the external DRAM #2.

The GOR Computing Pipeline performs 2D structure prediction of proteins, which consists of three sub-modules. The first one, Prediction, is used to predict preliminary secondary structure for each amino acid and the latter two stages for correction. The Data Buffers between adjoining stages are used for delivering middle results. Three computation modules perform the complete GOR algorithm procedure in data driven pipeline mode. When the preliminary secondary structure of sequence with ID #1 is generated by the Prediction module, it will be delivered to the next stage right now for result correction. The Prediction module will deal with the next sequence with ID #2. The result generated by the Correction1 (stage 2) module will be delivered to the Correction2 by the Middle correction result buffer for final correction. The final prediction result generated by the Correction2 module is sent to the Data Back-writing Controller and written back to external DRAM. The first stage, predict module is augmented with a local memory that consists of two parts: a ROM module to store constants and a RAM block to store parameter tables. Both of them are implemented by on-chip Block RAMs. We also set some transitive registers between adjoining sub-modules, invisible here, to deliver reusable elements for pipelined computing.

### FPGA implementation

#### Lookup parameter table in parallel

The prediction of fundamental conformation for each amino acid involves looking up parameter tables (called *infor_pair* and *infor_dir* in the GOR-IV package) to get the conditional probability values obtained from experimental methods. The tables are addressed by the residue type and relative position of amino acids in current window with a fixed width of 17 residues.

To calculate three probability values of fundamental structure of current amino acid, we first converse the address according to the residue type and relative position in computing window, then lookup the tables to obtain the probability values. The number of query operations for predicting 2D structure of a protein with length *N* is 272*×N* and only one valid parameter is read back for computing for each query operation. Additionally, the address interval between adjacent two query operations equals 21*×*21*×*136*×*8 Byte in *infor_pair* table and the query order has no influence on final prediction result since no data dependency exists in address conversion. The serial look-up order implemented in the GOR-IV software results in a great deal of small granularity discontinuous memory access operation and Cache missing. For parallel computation, centralized tables will become the performance bottleneck. We have to distribute the original parameter table (about 468 KB) to 136 parts, each of which is stored in an on-chip Block RAM module with dual-port so that probability values can be read without memory conflict. All the storage modules are independent and 136 parameters can be read out per cycle. Additionally, we transform the raw data in probability tables from decimal fraction with double precision into integer reducing data width from 64-bit to 32-bit without affecting accuracy. The storage requirement and logic consumption is reduced by 50% and 75%, respectively. We also designed multi-level tree-like adder array to sum up 136 probability values in a pipeline mode.

#### The task pipeline

**Figure 2:**
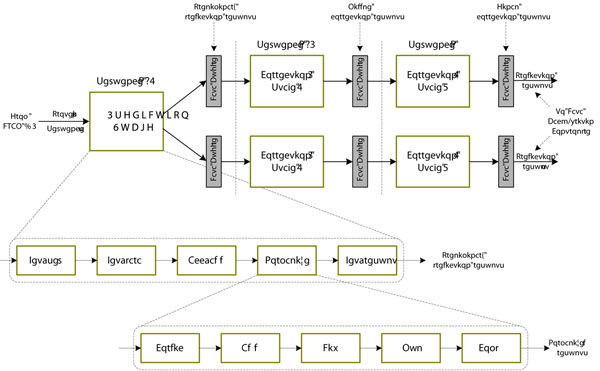
**The task pipeline in the GOR algorithm accelerator**. This figure describes the structure of the task pipeline in the GOR-IV algorithm accelerator. It is comprises a prediction stage and two correction stages. Multiple parallel correction modules are adopted to improve back-end processing capability.

Similar to the software version, the task pipeline in the GOR-IV algorithm accelerator also comprises a prediction stage and two correction stages as shown in Figure 2. We implemented a competed computing pipeline to accelerate the critical part, preliminary prediction, in the GOR program since most of the execution time, more than 99%, is spent in this stage. The core of the preliminary prediction module is composed of five steps: Get_seq (get amino acid code in protein sequence), Get_para (lookup parameter table and get probability values of fundamental structure in parallel), Acc_add (sum up multiple probability values using multi-level tree-like adder array in a pipeline mode), Normalize (compute the corresponding probability values of three fundamental conformation and selects the largest one), Get result (judge the fundamental conformation for each amino acid). The Normalize module is also composed of five sub-steps: Cordic (implement exponential operation), Add, Div, Mul and Comp (implement 32-bit float addition, division, multiplication and comparison operation, respectively). The whole preliminary prediction module is decomposed by 109 pipeline levels and the prediction results are stored in the Data Buffers for back-end correction processing.

Our experimental results show that the execution time of the three stages in the GOR algorithm accelerator is basically balanced generally. The first stage has powerful computing capability as the parallel table look-up strategy is adopted and there is no obvious performance bottleneck in our pipeline architecture because the balance of pipeline levels was carefully considered. Moreover, the overhead of the prediction stage for an input protein sequence with length *N* is fixed. However, because of the uncertain execution time of back-end processing for correction, which is closely related to the fundamental conformations in preliminary prediction results, and the serial correction procedure (only one fundamental conformation can be computed per cycle), the back-end process capability can’t catch up with the throughput of the prediction unit.

To solve the problem, we adopt the multi-channel parallel correction method by duplicating several back-end correction modules as shown in Figure 2. Because each correction module accesses the Data Buffer contemporarily to get the amino acid characters and corresponding 2D conformations, several Data Buffers copies are set to buffer preliminary and middle results for post processing. Thus, multiple sequences can be corrected in parallel. The final prediction results generated by each back-end process module are stored in individual Data Buffer and written back to the external DRAM #2 with FCFS (First Come First Serve) strategy by the controlling of the Data Back-writing Controller.

**Figure 3:**
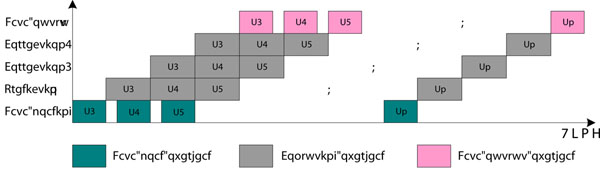
**The space-time diagram of the task pipeline in the GOR algorithm accelerator.** This figure gives another view of the parallel execution of the task pipeline in the GOR algorithm accelerator. *S*1, *S*2, ..., *Sn* represent the sequence identifications in protein database. The overhead for data loading and output is fully overlapped with pipelined computation accept for the overhead for loading the first sequence.

Figure 3 gives another view of the parallel execution of the task pipeline in the GOR algorithm accelerator. *S1, S2, ..., Sn* represent the sequence identifications in protein database. In the execution space-time diagram, we observe that there is no obvious load imbalance among multiple processing stages. The pause overhead for synchronizing multiple tasks is small compared with computation time, and the overhead for data loading and output is fully overlapped with pipelined computation accept for the overhead for loading the first sequence.

## Results and discussion

### Experimental environment

We implement the fine-grained parallel GOR-IV accelerator in an FPGA testbed. The testbed is mainly composed of one large scale FPGA chip, Virtex5 XC5VLX330 from Xilinx, two 2GB DDRII SODIMM modules, KVR 667D2S5/2G from Kingston and a PCI-E*×*8 interface (implemented by V5 XC5VLX50T) to the host computer. Specifically, our accelerator has the capability of global dynamic reconfigurability, which can be reconfigured in 60ms. This means that the configuration efficiency is improved by 2*~*3 orders of magnitude compared to the conventional configuration methods using a JTAG cable or other platform configuration device for identical design. The software for protein 2D structure prediction, GOR-IV, developed by J.Garnier, D.Osguthorpe and B.Robson (downloaded from the website http://abs.cit.nih.gov/gor/), runs on a desktop computer with AMD Quad 9650 2.3GHz CPU and 3.0GB Memory at level O3 compiler optimization. We also measure the software execution time on Intel Core2 Duo E7400 CPU platform to verify the acceleration of our approach.

### FPGA resource usage

We place only one GOR algorithm accelerating engine on FPGA chip, XC5VLX330 from Xilinx, to evaluate the resource usage. As shown in Table 1, each accelerating engine consumes 10040 Slices, 2 DSP48E modules and 159 Block RAMs (36Kbits each), sum to 5088 Kbits of memory, more than 55% (most of the them are consumed by implementing parameter table for protein prediction in stage1). However the proportion of logic resource used is only 19%. Thus, the critical constraint for accelerating the GOR package on FPGA chip is not logic resource but memory capacity. Experimental results show that our implementation can reach a clock frequency of over 160MHz (post-place & route not synthesize frequency) since there is no obvious performance bottleneck in our computing pipeline because of the balance of multiple processing stages was carefully considered and the IO overhead for external DRAM access will not become the limiting factor. The synthetical results also show that at least six GOR accelerating engines can be fitted on the new generation FPGA chip with largest memory capacity, XC6VSX475T currently. Assuming that hardware continues to improve by the Moore’s law, it is estimated that more accelerating engines will fit on a single FPGA chip and more remarkable speedup can be achieved in two years.

**Table 1:** FPGA utilization summary

FPGA	Engines	Slices /(%)	Memory /(%)	DSP /(%)	Frequency
XC5VLX330	One	10040 /(19%)	159 /(55%)	2 /(1%)	166 MHz
XC6VSX475T	Six	49880 /(67%)	932 /(88%)	12 /(1%)	166 MHz

### Performance compared to the GOR-IV software

We verify the correctness of our implementation in two steps. First, we verify the functional correctness of the hardware using software emulation with ModelSim SE 6.2h EDA tool. Then, we scan protein database on our testbed to compare the computational results with the ones produced by pure software to verify the correctness of the prediction results generated by our accelerator. We test four protein databases with different scale: gbenv6.fsa_aa (8,296 sequences, 1,772,705 amino acids), gbcon113.fsa_aa (10,774 sequences, 5,526,602 AAs), rsnc.0525.2010.faa (274,827 sequences, 88,448,646 AAs), and env_nr (6,028,192 sequences, 1,207,406,759 AAs), downloaded from the NCBI ftp server (ftp://ftp.ncbi.nih.gov/ncbi-asn1/protein_fasta), using hardware and software solutions respectively. The experimental results show that both the GOR-IV program and our fine-grained parallel GOR algorithm accelerator produce identical prediction results. To our knowledge, there is no parallel software version of the GOR package running on multi-processor or multi-core platforms and there is no parallel hardware implementation on FPGA or GPUs at present. Thus, we have to compare the performance to general-purpose CPUs.

### Speedup

Taking the AMD Phenom 9650 Quad CPU as the base, we compare the execution times and speedups among three different platforms, including two general-purpose computers and our GOR algorithm accelerator. The execution time on FPGA accelerator includes the computation time, the time for sending the protein databases and taking results back to the host for display. Despite the variation in CPU type, clock frequency, main memory capacity, second-level cache capacity and operate system versions, the two general-purpose computers exhibit similar performance for an input protein database. As shown in Table 2, the Intel E7400 CPU shows a slight advantage over AMD Phenom 9650, achieving about 1.3x~1.5x speedup. It partially because the frequency of E7400 CPU is higher than AMD Phenom 9650 and the current GOR package has not been parallelized based on multi-core processor platforms for an input protein sequence at present. Thus, the performance of the GOR package running on a Quad-CPU is similar to Dual-Core CPUs.

**Table 2:** Experiment results of execution time (s) and speedup (Sp) on different platforms for four protein databases with different scale.

Test Platforms	gbenv6.fsa_aa		gbcon113.fsa_aa		rsnc.0525.2010.faa		env_nr	
	Time	Speedup	Time	Speedup	Time	Speedup	Time	Speedup

AMD^(1)^	8.02	1	24.81	1	392.81	1	4308.66	1
Intel^(2)^	5.11	1.56	16.28	1.52	270.66	1.45	3161.54	1.36
FPGA^(3)^	0.12	66.8	0.14	177.2	0.91	430.6	9.81	439.2

The hardware experimental environment of different platforms are listed as follows.

AMD^(1)^: Phenom 9650 Quad CPU, 2.3GHz, 3.0GB memory;

Intel^(2)^: Core2 Duo E7400 CPU, 2.8GHz, 3.0GB memory;

FPGA^(3)^: XC5VLX330 FPGA chip, 166MHz, 4.0GB memory.

However, the FPGA accelerator exhibits a significant speedup over the GOR-IV package running on AMD Quad-Core CPU for four protein databases. From Table 2, we observed that the accelerations relative to software version range from 66.8-fold (for the gbenv6.fsa_aa) to 439.2-fold (for the largest PDB, env_nr). Even compared to the Intel Core2 Duo E7400 CPU, a speedup factor of more than 320x over the GOR-IV program can be achieved for the largest PDB with 6,028,192 sequences and 1,207,406,759 amino acids. Moreover, there is no data dependency in the multiple task of executing protein 2D structure prediction with different queries. Protein folding with multiple sequences, called inter-task parallelization or coarse-grained parallelization, can be performed independently. The only shared data are the parameter tables. Multiple sequence folding tasks can be distributed to multi-core CPU platforms to utilize computing resources efficiently. These implementations focus on database partitioning and load balance, instead of the parallelism of structure prediction for a single query. We implemented the multi-core GOR version with Multi-thread SIMD optimization on AMD Quad-Core CPU and four sequences can be executed in parallel at a time. We gain about 3.9x speedup over the naive GOR-IV version on quad-core CPU platforms. Thus, even compared to the optimized GOR version based on multi-core CPU, FPGA accelerator still achieved over 110x speedups for the largest PDB, env_nr. Furthermore, we can implement six accelerating engines on a FPGA chip XC6VSX475T and an average speedup of more than 600x can be achieved compared to the optimized GOR software implementation.

### Power consumption

We measured the average power consumption using a pincer galvanometer (equipment type HIOKI3290) in three steps. First, we measured the current consumed by the host PC on idle condition without FPGA accelerator. Second, we measured the current consumed by the entire prototype system configured with FPGA accelerator working in computing state. We can obtain the current used by the accelerator by subtracting the values from the two conditions. The power consumption can be computed using the expression of *voltage×current×power coefficient*.

We compare the power consumption ratio among the three different computing platforms. The average power consumption of the AMD Phenom 9650 and Intel Duo-core E7400 ranges from 65W to 95W [28]. However, our fine-grained GOR algorithm accelerator consumes about 20W as measured by a galvanometer, saving more than 70%. We also computed the entire system power consumption of protein structure predicting platform with or without FPGA accelerator by measuring the electrical current of host computer. Experimental results show that the average power consumption of the AMD platform is about 135W when running the original GOR-IV program. The average power consumption of calculating platform configured with one FPGA accelerator is less than 155W, increases only 15%, however, the computational power is improved by more than 400x. Hence, considering the high performance and low power consumption of FPGA chips for accelerating secondary prediction application of protein, we believe the application-specific fine-grained parallel accelerator prototype provides a promising solution over the general-purpose implementations.

## Conclusion and discussion

Proteins are complex macro-molecules that perform vital functions in biological systems. Nowadays, the protein folding problem has become a central research interest in modern molecular biology. Although the prediction of 3D structure is the ultimate goals, the 2D structure prediction from protein sequence is still a more feasible intermediate step in this direction. The GOR method based on information theory and Bayesian statistics is one of the earliest and most successful methods in 2D structure prediction. Although predicting protein 2D structure using the GOR approach is efficient in the classical sense, the execution time is still intolerable with the steep growth in protein database.

In this work, we propose a complete fine-grained parallel hardware implementation on FPGA to accelerate the GOR-IV package for 2D protein structure prediction. To improve computing efficiency, we partition the parameter table into small sections and access them in parallel. We aggressively exploit data reuse schemes to minimize the need for loading data from external memory. The whole computation structure is carefully pipelined in order to overlap sequence load, computing and back-writing operations as much as possible. We implemented the complete GOR desktop system based on a single FPGA chip (XC5VLX330). The experimental results show a factor of more than 430x over the original GOR-IV version and 110x speedup over the optimized version with multi-thread SIMD implementation running on a PC platform with AMD Phenom 9650 Quad CPU for scanning the whole protein database with 1,207,406,759 amino acids. Moreover, we estimated that an average speedup of more than 600x can be achieved on a FPGA chip XC6VSX475T by fitting six computing engines compared to optimized GOR version. However, the power consumption is only about 30% of that of current general-propose CPUs.

To the best of our knowledge, our design is the first FPGA implementation for accelerating the protein folding based on the GOR algorithm at present. Considering the significant performance improvement on FPGA chips, we believe that the application-specific fine-grained scheme implemented in our accelerator prototype provides significant advantages over general-purpose schemes for protein folding application. Moreover, our FPGA algorithm accelerator not only suggests a huge potential performance for parallelizing 3D structure prediction of protein but also can be applied to a desktop computing platform to resolve other large-scale bioinformatics and computational biology applications.

## Authors' contributions

Fei Xia carried out the fine-grained parallel GOR algorithm, participated in the characteristics analysis of the GOR algorithm and drafted the manuscript. Yong Dou conceived of the study, and participated in its design and helped to draft the manuscript. Guoqing Lei participated in the analysis of original GOR-IV package, hardware implementation and performance & power consumption measurement. YuSong Tan participated in the discussion of the study and correctness verification. All authors read and approved the final manuscript.

## Competing interests

The authors declare that they have no competing interests.
